# Optically isolated High-Speed data acquisition system for ion mobility spectrometry

**DOI:** 10.1016/j.ohx.2026.e00793

**Published:** 2026-05-22

**Authors:** Jannik Wuttke, Tim Kobelt, Jonas Winkelholz, Martin Lippmann, David Bailey, Stefan Zimmermann

**Affiliations:** Leibniz University Hannover, Institute of Electrical Engineering and Measurement Technology, Department of Sensors and Measurement Technology, Appelstr. 9A, 30167 Hannover, Germany

**Keywords:** Data acquisition, Optical isolation, High-speed, JESD204 interface, Ion mobility spectrometry, IMS

## Abstract

Many sensor applications require sensor data transmission from high electrical potential to ground potential. For example, the instrumental setup of ion mobility spectrometers (IMS) can be greatly simplified by operating the ion source at ground potential and the detector at high electrical potential. This operation mode demands the ion current signal to be transmitted electrically isolated from the detector to the data acquisition system. Furthermore, many applications require high sampling rate and high resolution of the data acquisition. Therefore, a novel open-source high-speed data acquisition system is presented that enables optically isolated data acquisition and facilitates a signal-to-noise ratio (SNR) of up to 87.5  dB with an adjustable sampling rate of up to 12.5 MS/s. To minimize the number of communication lines, an analog–digital-converter (ADC) with a serial high-speed interface is used instead of a parallel interface. Thereby, the communication lines can simply be isolated by only two common, low-cost optical network transceivers. In order to increase the SNR of the data acquisition, a commercial system on a chip (SoC) module digitally low-pass filters the ADC data and then reduces its sampling rate. An ethernet with TCP/IP connection is used to retrieve, visualize and save the recorded measurement data. In addition to the optically isolated high-speed data acquisition, the data acquisition system has one trigger pulse input channel and seven configurable pulse output channels, which enable compatibility with further control electronics.

## Graphical abstract

**Figure:**
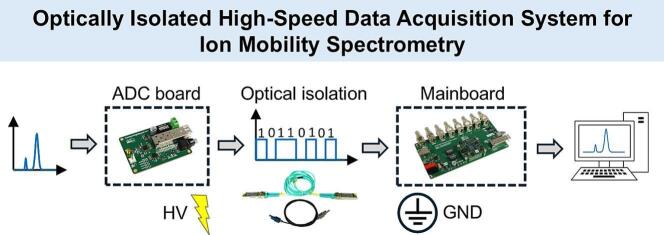

## 1 Specifications table

**Table:** 

Hardware name	*Optically Isolated High-Speed Data Acquisition System*
Subject area	• Engineering and materials science • Chemistry and biochemistry
Hardware type	• Measuring physical properties and in-lab sensors • Electrical engineering and computer science
Closest commercial analog	*CS548 Isolated Oscilloscope with CS1200 Remote Digitizer from cleverscope, for $ 17,230*
Open source license	*CC BY-SA 4.0*
Cost of hardware	*€ 1600*
Source file repository	*https://doi.org/10.17632/p7grnjmmcs.1*

## 2 Hardware in context

Ion mobility spectrometers (IMS) are analytical instruments used for detecting and identifying chemical compounds in the gas phase. The identification of different compounds is based on the ion specific drift velocities of the ionized compounds in an electric field in a neutral gas [1]. This method is particularly characterized by low detection limits in the ppt-range [2] and short response times of just one second [3]. Although IMS was originally developed for the detection of explosives, drugs of abuse and chemical warfare agents [4], they have become widely adopted in different applications, such as food analysis [5], exhaled breath analysis [6], environmental analysis [7], as a detector in gas chromatography (GC-IMS) [8] and as a further separation dimension in mass spectrometry [9]. Since many scientific questions remain unanswered, simplifying research through the publication of open-source IMS hardware is crucial [10], [11], [12], [13], [14]. As recent publications in IMS continue to show higher resolutions, ever higher demands are being placed on the sampling rate and resolution of the IMS data acquisition, especially for high kinetic energy ion mobility spectrometry (HiKE-IMS) [15], [16]. Furthermore, the instrumental complexity of an IMS is considerably reduced if the ion source can be operated at ground potential and the detector and the transimpedance amplifier at high electric potential [17]. Therefore, the open source IMS data acquisition system with pulse generation published by Kobelt et al. [18] was significantly enhanced in this work with a faster, optically isolated high-speed, high resolution data acquisition based on a faster analog-to-digital converter (ADC) and commercial system-on-a-chip-module (SoC-module). The pulse generation and the trigger input, developed by Kobelt et al, have been fully integrated now in the novel high-speed data acquisition system. As the high-speed data acquisition system is compatible with the open-source “IMS Control” software provided by Kobelt et al. [19], the data acquisition system can be configured via a computer. Especially the innovation in optically isolated data transmission should be emphasized: By using a high-speed ADC with a serial interface (JESD204) instead of a parallel interface for communication, the number of data communication lines to be electrically isolated is minimized. Therefore, isolated data transmission can be easily realized by using two commonly used low–cost optical transceivers from network technology (SFP + modules).

Commercially available data acquisition systems (e.g. Keysigth DSOX2004A) have high sampling rates (2 GS/s), but are not electrically isolated, are expensive (€ 2953) and have low resolutions [20]. Electrically isolated probes for oscilloscopes also exist, but these are usually expensive (e.g. Tektronix, IsoVu^TM^ isolated voltage probes, € 12,900 [21]).

A technically comparable commercial alternative for an isolated high-speed data acquisition system is, for example, the CS548 isolated oscilloscope with CS1200 remote digitizer from cleverscope. Table 1 shows a comparison of the key specifications between the optically isolated high-speed data acquisition system presented here and the CS584 isolated oscilloscope with CS1200 remote digitizer from cleverscope. It should be noted that the CS1200 offers far more features justifying its higher costs.

**Table 1:** Comparison of the key specifications between the optically isolated high-speed data acquisition system presented here and the CS584 isolated oscilloscope with CS1200 remote digitizer from cleverscope.

	Optically Isolated High-Speed Data Acquisition System for Ion Mobility Spectrometry	CS548 isolated oscilloscope with CS1200 remote digitizer from cleverscope [22]
Isolation voltage	tested to 13 kV	30 kV
Sampling rate	12.5 MSps	500 MSps
Resolution	16 bit	14 bit
SNR at 1 MHz	85.3 dBFS	64 dBc
Costs	*€ 1600*	$ 17,230

## 3 Hardware description

• *Data acquisition consists of two electrically isolated printed circuit boards, that enabled isolated data acquisition (tested up to 13 kV)*
• *High resolution (16 bit) with high signal-to-noise ratio (SNR) up to 87,5 dB (14.2 bit effective number of bits (ENOB)) by up to 12.5 MS/s*
• *Using high-speed ADC with serial high-speed interface enables easy electrically isolated data transmission with low-cost, optical transceivers*
• *Gaining SNR by using a low-pass finite-impulse-response (FIR) filter in combination with downsampling*
• *Trigger input and seven pulse output channels to control additional electronics (*e.g. *ion gates)*
• *Visualizing and storing data via the open source software “IMS Control”*

**Fig. 1:**
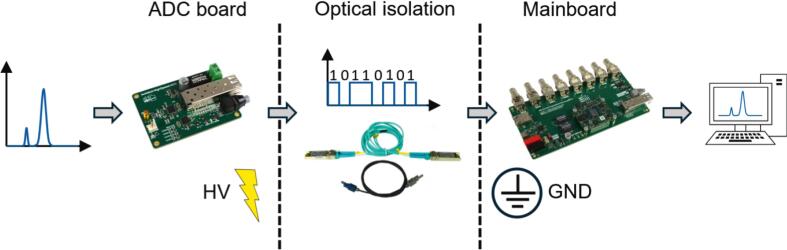
Concept of the optically isolated high-speed data acquisition system.

**Fig. 2:**
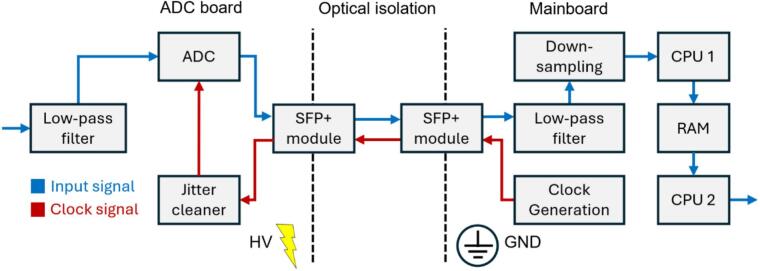
Block diagram of the optically isolated data acquisition.

In IMS, the analog input voltage of the data acquisition, which is usually the output voltage of a transimpedance amplifier, is often at high electrical potential [17]. In order to acquire this voltage despite the high electrical potential of the transimpedance amplifier, the data acquisition must be electrically isolated. Therefore, the high-speed data acquisition system presented here (Fig. 1) consists of two electrically isolated PCBs called ‘ADC board’ and ‘mainboard’. Once the analog input voltage has been digitized by an analog-to-digital converter (ADC) on the ADC board, the digitized input voltage is transmitted to the mainboard via optical transceivers and associated optical fibers. Due to the electrically isolated communication via optical fibers, the ADC board can be operated at high electrical potential and the mainboard at ground potential. By using a high-speed ADC with a serial interface (JESD204) instead of a parallel interface for communication, the number of data communication lines is minimized. This allows the optical data transmission of the digitized input voltage to be implemented easily at low-cost using two commonly used optical transceiver from network technology (SFP + modules). The digitized input voltage received by the mainboard is then digitally filtered and stored on the mainboard. By connecting the mainboard to a PC via an ethernet cable, either via a local network or a point-to-point connection, the digitized input voltage can be visualized online and stored on the PC using the “IMS Control” software published by Kobelt et al. [19]. Additionally, with the “IMS Control” software the data acquisition system can also be configured regarding the duration of recording and sampling rate. With regard to the sampling rate, four predefined options are available: 12.5 MS/s, 10 MS/s, 5 MS/s and 4 MS/s. Following the description of the concept of the optically isolated high-speed data acquisition system, a more detailed representation of the data acquisition system is provided in the following text and in the block diagram (Fig. 2).

**Fig. 3:**
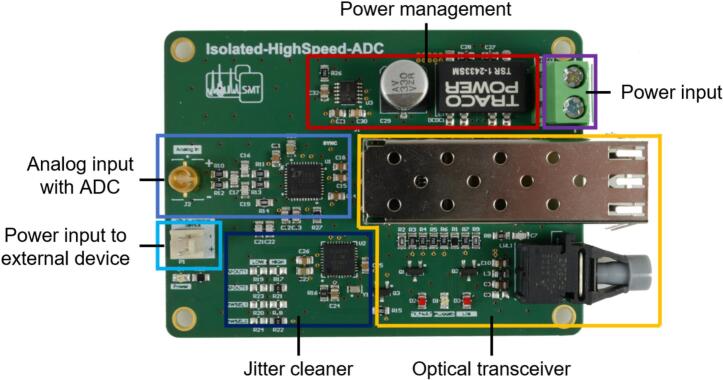
Overview of the ADC board.

The analog input voltage, normally the output voltage of a transimpedance amplifier, must be transmitted to the ADC board (Fig. 3) via a differential coaxial connector (Samtec, CJT-T-P-HH-ST-TH1) to ensure signal integrity. Reducing aliasing effects, the analog input voltage is then limited in bandwidth by a first-order passive low-pass filter and sampled by the ADC (Analog Devices, LTC2274) with 16 bits at a sampling rate of 100 MS/s. It should be noted that this initial slight low-pass filtering for antialiasing is sufficient, as the sampling rate of 100 MS/s is very high in relation to the maximum bandwidth of a few MHz reached in HiKE-IMS. Furthermore, the digitized input voltage is then sharply digitally low-pass filtered on the mainboard. Since the transfer functions of all components in the signal flow are multiplied, it must be ensured that the cut-off frequency of the transimpedance amplifier is always less than the cut-off frequency of the data acquisition system, to avoid losing any signal information through the data acquisition system. In order to comply with the input voltage range of the ADC, the differential input signal, respectively the output voltage of the transimpedance amplifier, must have a common mode voltage of 1.25  V and a maximum analog input voltage difference of 1.125  V. Therefore, it is recommended to adapt the analog input voltage to this voltage range, for example, by using a fully differential operational amplifier as output stage of the transimpedance amplifier in the IMS. It should be noted that even low ohmic resistance values in the output stage of the fully differential operational amplifier have a considerable effect on the on the cut-off frequency of the passive low-pass filter.

The digitized input voltage is then transmitted from the ADCs serial high-speed interface to the optical transceiver (small form-factor pluggable module, SFP + module). In addition to the SFP + module, a DC-capable optical receiver (Broadcom, AFBR-2624Z) is used, which is essential for the synchronization of the communication of the serial high-speed interface. In addition to the transmission of the digitized input voltage, the SFP + module also receives the 100 MHz clock signal transmitted from the mainboard as reference for the 100 MS/s ADC sampling. Since the clock jitter $\sigma_{Jitter}$, especially at high sampling rates, decreases the maximum possible ${\mathrm{SNR}}_{max}$, it must be minimized. The maximum possible ${\mathrm{SNR}}_{max}$ of an ADC at a given input frequency $f_{in}$ and a clock jitter $\sigma_{Jitter}$ is calculated by equation (1) [23]:

(1) $${\mathrm{SNR}}_{max}=-20\log_{10}\left(2\pi ∙f_{\mathrm{in}}∙\sigma_{\mathrm{Jitter}}\right)$$

In order to minimize the clock jitter $\sigma_{Jitter}$, a jitter cleaner (Skyworks, SI5317) is used, which guarantees a jitter of less than 300 fs. With a maximum possible input frequency $f_{\mathrm{in}}$ of 6.25 MHz, which is the Nyquist frequency of the highest predefined sampling rate, this results in a maximum possible ${\mathrm{SNR}}_{max}$ of 98.6 dB. Therefore, the noise power added by the clock jitter can be neglected in relation to the noise power added by the ADC itself with a ${\mathrm{SNR}}_{ADC}$ of 77.6  dB.

The power input of the isolated ADC board can be connected to a power supply via a terminal block. By using a buck converter (Traco, TSRN 1-2433SM), the board can be flexibly supplied with a wide supply voltage between 5 V and 24 V. Besides the optically isolated communication lines, the power supply must be isolated either to operate the ADC board at high electrical potential. The isolated power supply must provide 3  W and can either be realized via a battery or an isolated DC/DC converter, which must be selected depending on the electrical potential to be isolated. Whereas the reference potential of a battery can be set to any electrical potential, the battery only has a finite electrical capacity. However, the use of an isolated DC/DC converter guarantees a continuous power supply, but DC/DC converters have finite electrical isolation. For example, a recommended isolated DC/DC converter for an isolation of up to 3  kV (continuously) is the Recom REC6–2405SRW/R10/A. For higher isolation of up to 50  kV continuously, the open-source isolated DC power supply from Hitzemann et al. is recommended [24].

In order to supply additional external PCBs with power (e.g. transimpedance amplifier), the pins of the power supply input terminal block are directly connected to the pins of the PSK connector.

**Fig. 4:**
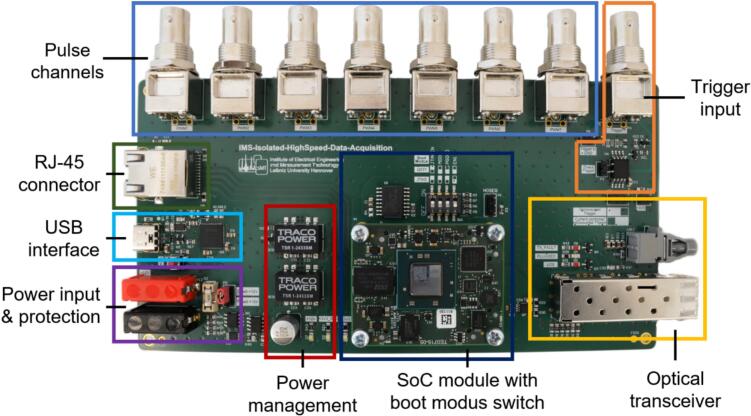
Overview of the mainboard.

On the mainboard (Fig. 4), a system-on-a-chip module (SoC module, Trenz, TE0715-05-73E33-A) with an AMD Zynq™ 7030-3E SoC was used to enable the communication with both the ADC board via the optical transceivers and a PC via ethernet with TCP/IP. The SoC module generates and transmits the 100 MHz clock signal via the SFP + modules to the ADC board for a 100 MS/s sampling rate. Furthermore, the SoC module receives the digitized input voltage from the ADC board and synchronizes the communication via a DC-capable optical transmitter.

Since even HiKE-IMS applications with a bandwidth of a few MHz does not require the full sampling rate $f_{s}$ of 100 MS/s, the principle of oversampling can be used to improve the SNR of the data acquisition [25]. Therefore, the ADC data sampled at 100 MS/s can be low-pass filtered and downsampled in the field programmable gate array (FPGA) inside the SoC to enable four predefined sampling rates (12.5 MS/s, 10 MS/s, 5 MS/s and 4 MS/s).. In order to avoid aliasing effects during further sampling a low-pass FIR filter is implemented in the FPGA for each predefined sampling rate. An FIR structure with symmetric filter coefficients is chosen to ensure a linear phase response, thereby avoiding signal distortion. The filter is realized using cascaded digital signal processing (DSP) slices within the FPGA. By utilizing all available DSP slices in the FPGA, a maximum filter order of 349 and, consequently, a maximum attenuation of more than −90  dB at the stop frequency is achieved eliminating any potential aliasing effects. According to Nyquist, the stop frequency used for each filter is half the predefined sampling frequency. Based on the specified FIR design with symmetric filter coefficients, a filter order of 349 and an attenuation of more than −90 dB at the stop frequency and the highest possible pass frequency, the FIR filter coefficients were calculated by the MATLAB filter wizard. This low-pass filtering does not only reduce the bandwidth of the digitized input voltage, but also significantly reduces the noise power distributed over the frequency range, which increases the SNR. Under the simplified assumption that the SNR_ideal_ of an ideal ADC is only composed of the quantization noise, which is evenly distributed over the frequency range, the SNR is increased by the oversampling ration OSR according to equation (2) [25]:

(2) $$\mathrm{SNR}={\mathrm{SNR}}_{ideal}+10∙\log_{10}\left(\frac{f_{s}}{2∙f_{\mathrm{LP}}}\right)={\mathrm{SNR}}_{ideal}+10∙\log_{10}\left(OSR\right)$$

Consequently, it is possible to trade high sampling rate for higher SNR. Furthermore, a low-pass FIR filter with symmetrical filter coefficients ensures a linear phase response. As this delays all frequency components of the sampled signal by the same amount of time, the signal is not deformed by the low-pass filtering [26]. The low-pass filtered and downsampled digitized input voltage is then stored in random-access memory (RAM) by central processing unit (CPU) 1 and can be retrieved by CPU2 from a client, like the “IMS Control” software, via a computer via ethernet with TCP/IP[19].

. Since the data acquisition is designed to be integrated in an IMS system, which compromises many further electronic components, such as driver electronics for ion sources and ion gates, we have integrated seven output trigger channels on the mainboard. Each output trigger channel is synchronized to the IMS data acquisition period and can be configured regarding its pulse delay and pulse width. Furthermore, the mainboard features a configurable trigger input that enables the data acquisition system to be coupled with further measurement devices, such as a laser, gas chromatograph or mass spectrometer. Since both the trigger output channels and the trigger input are identical to Kobelt et al. [18], no further description and characterization is given here. The power input of the mainboard can be connected to a power supply (minimum 5  W) via two 4  mm connectors. Ensuring safe operation, the power supply can be protected by a fuse and an undervoltage/overvoltage protection circuit designed for 12  V input voltage, described by Kobelt et al. [18]. If no additional current limitation is required and this is already provided by the power supply, the fuse and the protection circuit can also be disabled using a jumper. By disabling the fuse and the protection circuit, a wide input range for the power supply from 4.6  V – 42  V can be used according to the data sheet of the buck converter (Traco, TSRN 1-2433SM). In addition to the USB interface, which is used for programming and debugging, the mainboard has an RJ-45 connector, which enables ethernet with TCP/IP communication with a PC running the ‘IMS Control’ software [19]. As the SoC module has already integrated most of the required peripheral electronics, only the required boot mode needs to be set externally. Depending on the setting of the dip switch, the SoC on the SoC module boots either via JTAG and the USB interface, or via QSPI from a flash [27]. Furthermore, the mainboard also has several LEDs to simplify debugging.

## 4 Design files summary

**Table:** 

Design file name	File type	Open source license	Location of the file
ADC_Board.pdf	Schematics of the ADC board	*CC BY-SA 4.0*	*Mendeley Data*
*Mainboard.pdf*	Schematics of the mainboard	*CC BY-SA 4.0*	*Mendeley Data*
*Gerber_and_Drill_Files.zip*	Production files of the PCBs of the ADC board and the mainboard	*CC BY-SA 4.0*	*Mendeley Data*
*PCB-Projects.zip*	Altium Designer PCB Projects of the ADC board and the mainboard	*CC BY-SA 4.0*	*Mendeley Data*
*Firmware.zip*	FGPA and microcontroller firmware source code for Vivado/Vitis Classic for Xilinx Zynq	*CC BY-SA 4.0*	*Mendeley Data*
*BOOT.BIN*	Firmware binary for programming the SoC module	*CC BY-SA 4.0*	*Mendeley Data*
*fsbl.elf*	First stage bootloader for programming the SoC module	*CC BY-SA 4.0*	*Mendeley Data*
*Supporting_Information_Programming.zip*	Supporting Visualization for Programming the SoC module	*CC BY-SA 4.0*	*Mendeley Data*

## 5 Bill of materials summary

### 5.1 Mainboard

**Table:** 

Designator	Component	Number	Cost per unit	Total cost	Source of materials
	Mainboard PCB	*1*	*€ 127,00*	*€ 127,00*	*https://portal.multi-circuit-boards.eu/*
	SoC module	*1*	*€ 909,16*	*€ 909,16*	*https://www.trenz-electronic.de/de/SoC-Modul-mit-AMD-Zynq-7030-3E-1-GByte-DDR3L-32-MByte-Flash-4-x-5-cm/TE0715-05-73E33-A*
C1, C18, C23, C26, C27, C31, C32, C19, C20, C21, C22, C35	Capacitor SMD 0805 10uF X5R	12	*€* 0,13	*€ 1,56*	https://eu.mouser.com/ProductDetail/Murata-Electronics/GRM21BR6YA106KE43L?qs = K0Sa7bCb58In4wCNhrzhRQ%3D%3D
C2, C4	Capacitor SMD 0805 1uF X7R	2	*€* 0,28	*€ 0,56*	https://eu.mouser.com/ProductDetail/KEMET/C0805C105K5RACTU?qs = iP0bYSAMAFrBrUflcErrLQ%3D%3D
C3, C5, C6, C7, C8, C9, C10, C12, C13, C14, C25, C29, C33	Capacitor SMD 0805 100nF X7R	13	*€* 0,09	*€* 1,17	https://eu.mouser.com/ProductDetail/YAGEO/AC0805KKX7R0BB104?qs = sGAEpiMZZMukHu%252BjC5l7YWFdFy%252Bfu6GW0wnreysloV8%3D
C11, C30	Capacitor SMD 0805 100pF NP0	2	*€* 0,19	*€* 0,38	https://eu.mouser.com/ProductDetail/KYOCERA-AVX/08051A101GAT2A?qs = sGAEpiMZZMsh%252B1woXyUXj75AxcN1UwS45QjnHNeH16I%3D %3D
C15	Capacitor SMD 0805 12nF NP0	1	*€* 0,23	*€* 0,23	https://eu.mouser.com/ProductDetail/Murata-Electronics/GCM2195C1H123JA16D?qs = ui%252B2d9lVEI5OMCZUvQmI2g%3D%3D
C16, C17	Capacitor SMD 0805 10pF NP0	2	*€* 0,29	*€* 0,58	https://eu.mouser.com/ProductDetail/Murata-Electronics/GQM2195C2E100GB12D?qs = 0JbGSuRBwBOH8VtXYaOf7A%3D%3D
C24, C28	Capacitor SMD 0805 22uF X7R	2	*€* 0,27	*€* 0,54	https://eu.mouser.com/ProductDetail/Murata-Electronics/GRM21BZ71A226ME15L?qs = sGAEpiMZZMsh%252B1woXyUXjwuZcO7kv9Ieo7tykp6AZL4%3D
C34	Capacitor SMD 330uF aluminium electrolytic	1	*€* 0,63	*€* 0,63	https://www.mouser.de/ProductDetail/Chemi-Con/EMZR350ARA331MHA0G?qs = sGAEpiMZZMtZ1n0r9vR22cpFQt4dfSbqdsiOa4wdjdTKhGySS%2FPE2Q%3D%3D
C36	Capacitor SMD 0805 2.2nF NP0	1	*€* 0,22	*€* 0,22	https://eu.mouser.com/ProductDetail/Murata-Electronics/GRM2165C1H222JA01J?qs = e8vIWuFTP5EQBM%2F%252BTMoeUA%3D%3D
D1	Zener Diode SMD(BZX84Z) 5 V1	1	*€* 0,1	*€* 0,1	https://www.mouser.at/ProductDetail/Nexperia/BZX84-C5V1235?qs = me8TqzrmIYW5nRjNejt%2Fyg%3D%3D&srsltid = AfmBOooHk03kw6UUe4-fFVF44Ia94sS0G-2pEffrxz-c3LXxv5r3KW2s
D2, D3, D7	LED SMD 0805 yellow	2	*€* 0,38	*€* 0,76	https://eu.mouser.com/ProductDetail/Kingbright/AP2012YD?qs = sXafwHSx%252BU%2FMO4ENADNdDQ%3D%3D
D4	LED SMD 0805 green	1	*€* 0,20	*€* 0,2	https://www.mouser.de/ProductDetail/Kingbright/APHCM2012CGCK-F01?qs = qqKal61G0KznDDeOGiLi0g%3D%3D
D5, D6	LED SMD 0805 red	1	*€* 0,30	*€* 0,3	https://www.mouser.de/ProductDetail/Kingbright/APTD3216LSURCK?qs = sGAEpiMZZMv0DJfhVcWlK8AiAHwfAGPKDd5wjoSpm06%2F78WU7m%2FISw%3D%3D
DCDC1, DCDC2	DCDC Converter (TSRN 1–2433SM)	2	*€* 13,71	*€* 27,42	https://de.rs-online.com/web/p/schaltregler/7553447
IC1	UV/OV Protection Controller (LTC4368)	1	*€* 7,66	*€* 7,66	https://www.mouser.de/ProductDetail/Analog-Devices/LTC4368IMS-2PBF?qs = 5aG0NVq1C4yQGBsRaTKLEg%3D%3D
*J1, J2*	*Connector LSHM-150*–*04.0-L-DV-A-S-K-TR*	2	*€* 7,61	*€* 15,22	https://eu.mouser.com/ProductDetail/Samtec/LSHM-150–04.0-L-DV-A-S-K-TR?qs=PB6%2FjmICvI0kfKxky75CoQ%3D%3D
J3	*Connector* LSHM-130–04.0-L-DV-A-S-K-TR	1	*€* 4,83	*€* 4,83	https://eu.mouser.com/ProductDetail/Samtec/LSHM-130–04.0-L-DV-A-S-K-TR?qs=PB6%2FjmICvI3ns1KYUMaNlw%3D%3D
J4	SFP + Connector SFPK-SL	1	*€* 3,47	*€* 3,47	https://www.mouser.de/ProductDetail/Samtec/SFPK-SL-TR?qs = rU5fayqh%252BE1PTVdlEqQZnQ%3D%3D
L1, L2	Chip bead, 220 Ohms, 0603	2	*€* 0,09	*€* 0,18	https://www.mouser.de/ProductDetail/TDK/MPZ1608S221ATA00?qs = pLY5GE0xrmLZeKkazChaNg%3D%3D
L3, L4, L5	Inductor SMD 0805 10uH	3	*€* 0,3	*€* 0,9	https://www.mouser.de/ProductDetail/ABRACON/ASMCI-0805–100 M−T?qs = sGAEpiMZZMukHu%252BjC5l7YY9R5kV26JimBpWi5g1l9Dk%3D
LWL1	Fibre Optic Transmitter AFBR-1624Z	1	*€* 18,78	*€* 18,78	https://eu.mouser.com/ProductDetail/Broadcom-Avago/AFBR-1624Z?qs = 60YbxKv0rtAaXQ7gKlmQcw%3D%3D
P1, P7, P9	Header 3 Pin	3	*€* 0,11	*€* 0,33	https://eu.mouser.com/ProductDetail/Wurth-Elektronik/61300311121?qs=PhR8RmCirEYxRWFJzNKsUw%3D%3D
P2	Ethernet Connector RJ45 10/100/1000BASE-T	1	*€* 13,33	*€* 13,33	https://eu.mouser.com/ProductDetail/Wurth-Elektronik/7498111120AR?qs = 1Kr7Jg1SGW%252ByNilysD1%2FJw%3D%3D
P3, P4, P8	Header 2 Pin	3	*€* 0,11	*€* 0,33	https://eu.mouser.com/ProductDetail/Wurth-Elektronik/61300211121?qs = t4813l51qx%252B1A5GYDQxPlw%3D%3D
P5, 'P10_PWM_Output1, P10_PWM_Output2, P10_PWM_Output3, P10_PWM_Output4, P10_PWM_Output5, P10_PWM_Output6, P10_PWM_Output7	BNC Socket	8	*€* 4,45	*€* 35,6	https://de.farnell.com/multicomp-pro/13–60-6-dgz/buchse-bnc-right-angle/dp/1712355
P12	4 mm banana plug red	1	*€* 2,92	*€* 2,92	https://eu.mouser.com/ProductDetail/Altech/973582101?qs = qOhVvio4tz6SzhhLDilKhw%3D%3D
P13	4 mm banana plug black	1	*€* 2,92	*€* 2,92	https://eu.mouser.com/ProductDetail/Altech/973582100?qs = qOhVvio4tz7gdcxnb%2FguLA%3D%3D
Q1	Bipolar Transistor SMD BC846	1	*€* 0,10	*€* 0,1	https://eu.mouser.com/ProductDetail/onsemi/BC846BLT1G?qs = vNc2DXHODiJuwwGLruzsRg%3D%3D
Q2, Q3, Q4	N-channel MOSFET SMD IRLML6346	3	*€* 0,35	*€* 1,05	https://eu.mouser.com/ProductDetail/Infineon-Technologies/IRLML6346TRPBF?qs = 9%252BKlkBgLFf3TGIqhHXU%2FwA%3D%3D
Q5, Q6	N-channel MOSFET SMD IRF8714	2	*€* 0,77	*€* 1,54	https://eu.mouser.com/ProductDetail/Infineon-Technologies/IRF8714TRPBFXTMA1?qs = 1Kr7Jg1SGW97Au2Wo%252B1AuQ%3D%3D
R1, R2, R3, R5, R6, R7, R8, R12, R22, R28	Resistor SMD 0805 10 K	10	*€* 0,04	*€* 0,4	https://eu.mouser.com/ProductDetail/YAGEO/SR0805KR-0710KL?qs = ABvmyp11KkjfiAMN9pb25w%3D%3D
R9, R10, R39	Resistor SMD 0805 120R	3	*€* 0,09	*€* 0,27	https://eu.mouser.com/ProductDetail/YAGEO/RT0805FRE13120RL?qs = sGAEpiMZZMtlubZbdhIBIMaxnMKvEBNdVgVhELgHzTg%3D
R11, R37	Resistor SMD 0805 1 M1	2	*€* 0,22	*€* 0,44	https://eu.mouser.com/ProductDetail/YAGEO/RC0805FR-131M1L?qs = sGAEpiMZZMtlubZbdhIBINSnPd898yDPkdUKGb%2F5eBg%3D
R13, R23	Resistor SMD 0805 2 K2	2	*€* 0,10	*€* 0,2	https://eu.mouser.com/ProductDetail/YAGEO/AF0805FR-0742K2L?qs = tggtontpCXMitgT1gluNTg%3D%3D
R14, R16	Resistor SMD 0805 5 K1	2	*€* 0,19	*€* 0,38	https://eu.mouser.com/ProductDetail/YAGEO/RC0805JR-105K1L?qs = qpJ%252B%252B%252Bdg6p0nSRKPOml2RA%3D%3D
R15	Resistor SMD 0805 12 K	1	*€* 0,22	*€* 0,22	https://eu.mouser.com/ProductDetail/YAGEO/RC0805FR-1312K1L?qs = m6lXFsvg5e0Zw8adAlq8vA%3D%3D
R17, R38	Resistor SMD 0805 1 K	1	*€* 0,09	*€* 0,09	https://eu.mouser.com/ProductDetail/YAGEO/AC0805DR-071KL?qs = sGAEpiMZZMtlubZbdhIBIMHZ5gvv1VGtgorNkL6aSNE%3D
R18	Resistor SMD 0805 300R	1	*€* 0,10	*€* 0,1	https://eu.mouser.com/ProductDetail/YAGEO/RC0805FR-7W300RL?qs=NgbZBzc1CyF6vHgHT%252BIDmg%3D%3D
R19, R20	Resistor SMD 0805 510R	2	*€* 0,09	*€* 0,18	https://eu.mouser.com/ProductDetail/YAGEO/RC0805FR-07510RP?qs = sGAEpiMZZMtlubZbdhIBIEgN9MQql2m6pN3%252B4ciI0x4%3D
R21, R44	Resistor SMD 0805 0R	1	*€* 0,1	*€* 0,1	https://eu.mouser.com/ProductDetail/Vishay-Draloric/RCA08050000ZSEC?qs = sGAEpiMZZMtlubZbdhIBICkZD26Dv0dBF8vbjjT8CPE%3D
R24, R26	Resistor SMD 0805 120R	2	*€* 0,9	*€* 0,18	https://eu.mouser.com/ProductDetail/YAGEO/RT0805FRE13120RL?qs = sGAEpiMZZMtlubZbdhIBIMaxnMKvEBNdVgVhELgHzTg%3D
R29, R30	Resistor SMD 0805 51R	2	*€* 0,24	*€* 0,48	https://eu.mouser.com/ProductDetail/Vishay/CRCW080551R1FKEAHP?qs = sGAEpiMZZMtlubZbdhIBILDMOtf4qnQou5fLRy8OaM0%3D
R31, R41, R42	Resistor SMD 0805 100R	3	*€* 0,7	*€* 0,21	https://eu.mouser.com/ProductDetail/YAGEO/RT0805BRB07100RL?qs=%2Ff7pOCXLR5c%2FAeqV6eMvJA%3D%3D
R32, R33, R34, R35, R36	Resistor SMD 0805 7 K5	5	*€* 0,17	*€* 0,85	https://eu.mouser.com/ProductDetail/Vishay/CRCW08057K50FKEAHP?qs = sGAEpiMZZMtlubZbdhIBIKMpMWeJoXcV9UcvR0VmUeM%3D
R40	Resistor SMD 0805 4 K7	1	*€* 0,23	*€* 0,23	https://eu.mouser.com/ProductDetail/Vishay-Draloric/RCS08054K75FKEA?qs=NKmfXavxMayPp7sSGctv1Q%3D%3D
R43	Resistor SMD 0805 1 K3	1	*€* 0,11	*€* 0,11	https://eu.mouser.com/ProductDetail/YAGEO/SR0805JR-7W1K3L?qs = sGAEpiMZZMtlubZbdhIBIEgN9MQql2m6wcndZ4BTc6E%3D
R45	Resistor SMD 0805 22 K	1	*€* 0,19	*€* 0,19	https://eu.mouser.com/ProductDetail/YAGEO/AR0805JR-0722KL?qs = tub3WQyKOl%2FfcisQcvcCNw%3D%3D
R46	Resistor SMD 0805 3 M9	1	*€* 0,09	*€* 0,09	https://eu.mouser.com/ProductDetail/YAGEO/RC0805FR-073M9L?qs = QrWOOBGzeCZ5p078sEy%2FdQ%3D%3D
R47, R48	Resistor SMD 0805 150 K	2	*€* 0,11	*€* 0,22	https://eu.mouser.com/ProductDetail/YAGEO/SR0805JR-47150KL?qs = xZ%2FP%252Ba9zWqZdliQ6zlXfPA%3D%3D
S1	Tactile Switch SMD TL3305BF260QG	1	*€* 0,17	*€* 0,17	https://eu.mouser.com/ProductDetail/E-Switch/TL3305BF260QG?qs = UrFqKgNWc7Tq35XCdypyiw%3D%3D
S2	154Series-FuseHolder SMD	1	*€* 2,02	*€* 2,02	https://eu.mouser.com/ProductDetail/Littelfuse/01550900 M?qs = DDoybve0rC%252BTXq1hqi5X1g%3D%3D
SW1	DIP switch SMD	1	*€* 1,18	*€* 1,18	https://eu.mouser.com/ProductDetail/Nidec-Components/CFS-0401 TB?qs = XeJtXLiO41TY9UNEWNjkYA%3D%3D
U1	EEPROM S25FL256SAGMFIR01	1	*€* 0,27	*€* 0,27	https://eu.mouser.com/ProductDetail/Microchip-Technology/24AA02E48T-I-OT?qs = h8kCHL4kpM5dzsPTuTrxSQ%3D%3D
U2	ESD protection diode SMD PRTR5V0U2X,215	1	*€* 0,62	*€* 0,62	https://eu.mouser.com/ProductDetail/Nexperia/PRTR5V0U2X215?qs = LOCUfHb8d9sDkgY4cRj8Lw%3D%3D&srsltid = AfmBOopDRQ_h9JKfBK6zN4uj0MqVp6osqTmt77wm5QegsMVNwBK2CRaH
U3	USB Interface IC FT2232H-56Q	1	*€* 4,47	*€* 4,47	https://eu.mouser.com/ProductDetail/FTDI/FT2232H-56Q-TRAY?qs = u4lROS522ZW4g0YyKZT0kg%3D%3D
U4	EEPROM 93AA56BT-I/OT	1	*€* 0,27	*€* 0,27	https://eu.mouser.com/ProductDetail/Microchip-Technology/93AA56BT-I-OT?qs = hul3WK2zl4p%252B6%2FsdRU7j4A%3D%3D
U5	USB C Connector 213716–0001	1	*€* 1,02	*€* 1,02	https://eu.mouser.com/ProductDetail/Molex/213716–0001?qs = IS%252B4QmGtzzqb95Au5%252B6Z%252BQ%3D%3D
U6	Optocoupler ACPL- K64L-560E	1	*€* 5,16	*€* 5,16	https://eu.mouser.com/ProductDetail/Broadcom-Avago/ACPL-K64L-560E?qs = KAs5CcNu0T5TSXsMeTh8Dg%3D%3D
U7	LVDS Interface IC SN65CML100DGKR	1	*€* 3,87	*€* 3,87	https://eu.mouser.com/ProductDetail/Texas-Instruments/SN65CML100DGKR?qs = sGAEpiMZZMutXGli8Ay4kMj2H7jHcXU%2FmlCRGiHwaO4%3D
XMER1	Common Mode Choke 744,233,670	1	*€* 1,26	*€* 1,26	https://eu.mouser.com/ProductDetail/Wurth-Elektronik/744233670?qs = E%2F%2FhvbtCqpMuo2paLccS6Q%3D%3D
Y1	Crystal 12 MHz	1	*€* 0,49	*€* 0,49	https://eu.mouser.com/ProductDetail/ABRACON/ABM8G-12.000MHZ-4Y-T3?qs = hxOQwdLlgepjgH1DpgEPEA%3D%3D

### 5.2 ADC board

**Table:** 

Designator	Component	Number	Cost per unit −currency	Total cost − currency	Source of materials
	ADC board PCB	*1*	*€ 71,85*	*€ 71,85*	*https://portal.multi-circuit-boards.eu/*
*C1, C4, C8, C11, C12, C13, C14, C15, C18, C24, C25, C26*	Capacitor SMD 0805 100nF X5R	*12*	*€ 0,09*	*€ 1,08*	https://eu.mouser.com/ProductDetail/YAGEO/AC0805KKX7R0BB104?qs = sGAEpiMZZMukHu%252BjC5l7YWFdFy%252Bfu6GW0wnreysloV8%3D
*C2, C5, C27*	Capacitor SMD 0805 22uF X7R	*3*	*€ 0,27*	*€ 0,81*	https://eu.mouser.com/ProductDetail/Murata-Electronics/GRM21BZ71A226ME15L?qs = sGAEpiMZZMsh%252B1woXyUXjwuZcO7kv9Ieo7tykp6AZL4%3D
*C3, C6, C9, C10, C28, C31*	Capacitor SMD 0805 10uF X5R	*6*	*€ 0,13*	*€ 0,78*	https://eu.mouser.com/ProductDetail/Murata-Electronics/GRM21BR6YA106KE43L?qs = K0Sa7bCb58In4wCNhrzhRQ%3D%3D
*C7*	Capacitor SMD 0805 100pF NP0	*1*	*€ 0,19*	*€ 0,19*	https://eu.mouser.com/ProductDetail/KYOCERA-AVX/08051A101GAT2A?qs = sGAEpiMZZMsh%252B1woXyUXj75AxcN1UwS45QjnHNeH16I%3D %3D
*C16, C19*	Capacitor SMD 0805 2.2nF NP0	*1*	*€ 0,22*	*€ 0,22*	https://eu.mouser.com/ProductDetail/Murata-Electronics/GRM2165C1H222JA01J?qs = e8vIWuFTP5EQBM%2F%252BTMoeUA%3D%3D
*C17*	Capacitor SMD 0805 1.5nF NP0	*1*	*€ 0,45*	*€ 0,45*	*https://eu.mouser.com/ProductDetail/KYOCERA-AVX/08055A152GAT2A?qs=%2FALOMWCg6cOgH5RYK6ae3g%3D%3D*
*C20, C23*	Capacitor SMD 0805 1uF X7R	*2*	*€ 0,28*	*€ 0,56*	https://eu.mouser.com/ProductDetail/KEMET/C0805C105K5RACTU?qs = iP0bYSAMAFrBrUflcErrLQ%3D%3D
*C21, C22*	Capacitor SMD 0805 3.3nF NP0	*2*	*€ 0,83*	*€ 1,66*	*https://eu.mouser.com/ProductDetail/KYOCERA-AVX/08055A332FAT2A?qs* *= o86%2F%2FV5KDpwUxsVk1RrDVA%3D%3D*
*C29*	Capacitor SMD 330uF aluminium electrolytic	*1*	*€ 0,63*	*€ 0,63*	https://www.mouser.de/ProductDetail/Chemi-Con/EMZR350ARA331MHA0G?qs = sGAEpiMZZMtZ1n0r9vR22cpFQt4dfSbqdsiOa4wdjdTKhGySS%2FPE2Q%3D%3D
*C30, C32*	Capacitor SMD 0805 4.7uF X7R	*1*	*€ 0,31*	*€ 0,31*	*https://www.mouser.de/ProductDetail/KEMET/C0805C475M9RACAUTO?qs* *= sGAEpiMZZMsh%252B1woXyUXj8cbwg7mY%252Bp%2FgX7%2FXe%2F2cog%3D*
*D1, D4*	LED SMD 0805 green	*2*	*€ 0,2*	*€ 0,4*	https://www.mouser.de/ProductDetail/Kingbright/APHCM2012CGCK-F01?qs = qqKal61G0KznDDeOGiLi0g%3D%3D
*D2, D3*	LED SMD 0805 red	*2*	*€ 0,3*	*€ 0,6*	https://www.mouser.de/ProductDetail/Kingbright/APTD3216LSURCK?qs = sGAEpiMZZMv0DJfhVcWlK8AiAHwfAGPKDd5wjoSpm06%2F78WU7m%2FISw%3D%3D
*DCDC1*	DCDC Converter (TSRN 1–2433SM)	*1*	*€ 13,71*	*€ 13,71*	https://de.rs-online.com/web/p/schaltregler/7553447
*J1*	SFP + Connector SFPK-SL	*1*	*€ 3,47*	*€ 3,47*	https://www.mouser.de/ProductDetail/Samtec/SFPK-SL-TR?qs = rU5fayqh%252BE1PTVdlEqQZnQ%3D%3D
*J2*	Differential coaxial connector CJT-T-P-HH-ST-TH1	*1*	*€ 12,21*	*€ 12,21*	*https://www.mouser.de/ProductDetail/Samtec/CJT-T-P-HH-ST-TH1?qs=PB6%2FjmICvI3dfW8RDpxn0g%3D%3D*
*L1, L2, L3*	Inductor SMD 0805 10uH	*3*	*€ 0,3*	*€ 0,9*	https://www.mouser.de/ProductDetail/ABRACON/ASMCI-0805–100 M−T?qs = sGAEpiMZZMukHu%252BjC5l7YY9R5kV26JimBpWi5g1l9Dk%3D
*LWL1*	Fibre Optic Receiver AFBR-2624Z	*1*	*€ 20,04*	*€ 20,04*	https://eu.mouser.com/ProductDetail/Broadcom-Avago/AFBR-2624Z?qs = 60YbxKv0rtDJIJtl89oIpg%3D%3D
*P1*	PSK Header 22–23-2021	*1*	*€ 0,14*	*€ 0,14*	https://www.mouser.de/ProductDetail/Molex/22–23-2021?qs = ILqg114nvd4YKlRlbo3yMg%3D%3D
*Q1, Q2, Q3*	N-channel MOSFET SMD IRLML6346	*3*	*€ 0, 35*	*€ 1,05*	https://eu.mouser.com/ProductDetail/Infineon-Technologies/IRLML6346TRPBF?qs = 9%252BKlkBgLFf3TGIqhHXU%2FwA%3D%3D
R1, 15	Resistor SMD 0805 120R	2	*€* 0,09	*€ 0,18*	https://eu.mouser.com/ProductDetail/YAGEO/RT0805FRE13120RL?qs = sGAEpiMZZMtlubZbdhIBIMaxnMKvEBNdVgVhELgHzTg%3D
R2, R9, R14	Resistor SMD 0805 100R	3	*€* 0,7	*€ 2,1*	https://eu.mouser.com/ProductDetail/YAGEO/RT0805BRB07100RL?qs=%2Ff7pOCXLR5c%2FAeqV6eMvJA%3D%3D
R3, R4, R5, R6, R7	Resistor SMD 0805 7 K5	5	*€* 0,17	*€ 0,85*	https://eu.mouser.com/ProductDetail/Vishay/CRCW08057K50FKEAHP?qs = sGAEpiMZZMtlubZbdhIBIKMpMWeJoXcV9UcvR0VmUeM%3D
R8	Resistor SMD 0805 1 M1	2	*€* 0,22	*€ 0,44*	https://eu.mouser.com/ProductDetail/YAGEO/RC0805FR-131M1L?qs = sGAEpiMZZMtlubZbdhIBINSnPd898yDPkdUKGb%2F5eBg%3D
R10, R11, R12, R13	Resistor SMD 0805 3R	4	*€* 0,10	*€ 0,4*	https://www.mouser.de/ProductDetail/Bourns/CR0805-J-3R0ELF?qs = sGAEpiMZZMtlubZbdhIBIPJRFm2x4gVbNEJ7c8HJdtE%3D
R15	Resistor SMD 0805 10 K	1	*€* 0,04	*€ 0,04*	https://eu.mouser.com/ProductDetail/YAGEO/SR0805KR-0710KL?qs = ABvmyp11KkjfiAMN9pb25w%3D%3D
R16, R21, R22, R27	Resistor SMD 0805 0R	4	*€* 0,1	*€ 0,4*	https://eu.mouser.com/ProductDetail/Vishay-Draloric/RCA08050000ZSEC?qs = sGAEpiMZZMtlubZbdhIBICkZD26Dv0dBF8vbjjT8CPE%3D
R26	Resistor SMD 0805 15 K	1	*€* 0,09	*€ 0,09*	https://eu.mouser.com/ProductDetail/YAGEO/RC0805JR-7W15KL?qs = tggtontpCXNqlYSAK9m2vA%3D%3D
S1	Terminal Block 1,729,128	1	*€* 0,79	*€ 0,79*	https://www.mouser.de/ProductDetail/*Phoenix*-Contact/1729128?qs = GFUSqQMLmmnCBVBY3dts9w%3D%3D&mgh = 1&vip = 1&utm_id = 20968985688&utm_source = google&utm_medium = cpc&utm_marketing_tactic = emeacorp&gad_source = 1&gad_campaignid = 20978868859&gclid=Cj0KCQiA7fbLBhDJARIsAOAqhsfjx0I9aJY5L4jpg0D7xRaoykKxCZ35bLZZQl-c9KS1-JXLhYC2j9saAm65EALw_wcB
U1	ADC LTC2274	1	*€* 183,54	*€ 1833,54*	https://www.mouser.de/ProductDetail/Analog-Devices/LTC2274IUJPBF?qs = hVkxg5c3xu8THpAPxeJ8Sw%3D%3D
U2	Jitter Cleaner SI5317C-C-GM	1	*€* 14,48	*€ 14,48*	https://www.mouser.de/ProductDetail/Skyworks-Solutions-Inc/SI5317C-C-GM?qs = p9T7GgSe1IFkWqeS9tzW%2FQ%3D%3D
U3	Linear Regulator LT3045	1	*€* 11,72	*€ 11,72*	https://www.mouser.de/ProductDetail/Analog-Devices/LT3045HMSETRPBF?qs = l7cgNqFNU1g2E5PK50Mk%2FA%3D%3D
Y1	Crystal 40 MHz ABM12W-40.0000MHZ-6-B1U-T3	1	*€* 0,86	*€ 0,86*	https://www.mouser.de/ProductDetail/ABRACON/ABM12W-40.0000MHZ-6-B1U-T3?qs = 5aG0NVq1C4wdQHdP%252BiWw6A%3D%3D

### 5.3 Connection cables

**Table:** 

Designator	Component	Number	Cost per unit −currency	Total cost − currency	Source of materials
	SFP + Transceiver	*2*	*€ 24,49*	*€ 48,98*	*https://www.alternate.de/TP-Link/TL-SM5110-SR-10Gbase-SR-SFPplus-LC-Transceiver/html/product/1702005*
	*Fiber patch cable LC*	*1*	*€ 5,59*	*€ 5,59*	*https://www.fs.com/de/products/88534.html?country* *= DE&currency = EUR&languages = Deutsch&paid = google_shopping&gad_source = 1&gad_campaignid = 17932539988&gclid=Cj0KCQiA7fbLBhDJARIsAOAqhsebwFPC_5Ta6aA5wIQtzE-ssLu_nRe8m4ntatR76rbmB-1Q7vfdtvcaAgaXEALw_wcB*

## 6 Build instructions

### 6.1 Safety measures

The assembly of the data acquisition presented here involves soldering components onto a PCB and may only be carried out by qualified specialist personnel. It must be taken into account that the heat generated during soldering can cause burns and harmful substances can be released and inhaled. Accordingly, both adequate protection for soldering work and sufficient ventilation must be provided.Although the data acquisition system itself does not contain voltages higher than 24  V, the system can be integrated in IMS systems, which have high voltages of up to several kV.. Only qualified personnel may work on high electrical voltages. All legal requirements and occupational safety regulations must be observed at all times.

### 6.2 Printed circuit board assembly

It is possible to either order a fully assembled PCB with all components based on the uploaded PCB files, or have only the PCB manufactured and solder the components yourself. In the case of self-assembly, it is recommended to use a stencil and solder paste for the surface-mounted devices (SMD). Small components should be assembled first. Due to a combined footprint, the trigger pulse channel and the trigger input can be equipped with either Bayonet Neill Concelman (BNC) socket or Sub-Miniature Version A (SMA) socket. Since the input protection and the trigger input described and characterized by Kobelt et al. [18] have good performance, they are integrated into this data acquisition. Consequently, the input protection used here also has an undervoltage protection of 7  V and an overvoltage protection of 14  V based on the resistance values R44 = 0  Ω, R46 = 3.9 MΩ, R47 = 150 kΩ and R48 = 150 kΩ. Furthermore, the trigger input can also be configured in three different operating modes [18]. By setting jumpers to P4 and P8, the “direct trigger” mode is enabled. As the trigger is connected directly to the input of the FPGA, it must be ensured that the trigger levels are 0  V and 3.3  V. However, if the application requires electrical isolation of up to 500  V of the trigger input, either the “optocoupled trigger” mode or the “current controlled optocoupled trigger” mode can be used. For “optocoupled trigger” mode, the jumper must be set to P7 via pins 3 and 2 and the resistors R19 and R20 must be adjusted according to Kobelt et al. depending on the input voltage, which enables trigger voltages of up to 24  V [18]. For “current controlled optocoupled trigger” mode the jumper must be set to P7 via pins 1 and 2, which enables input trigger voltages between 4  V and 34  V. However, it should be noted that the “current controlled optocoupled trigger” mode input has a voltage-dependent trigger delay [18].

### 6.3 Programming the data acquisition system

**Fig. 5:**
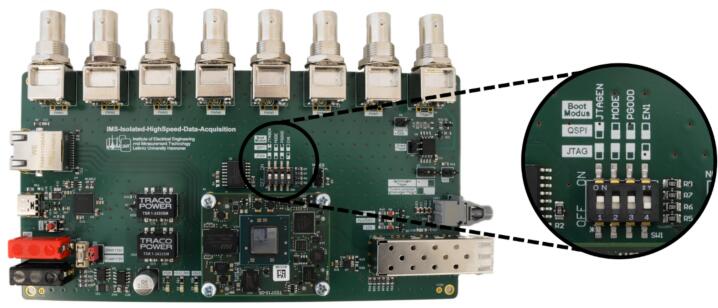
Boot modus configuration via dip switch.

To program the SoC module with the AMD Zynq™ XC7Z030, the dip switch SW1 must be set to JTAG boot mode according to the marking on the silkscreen as shown in Fig. 5. Therefore, the FT2232H-56Q (U3) must first be programmed on the mainboard, which enables a USB to JTAG interface for programming and a USB to UART interface for debugging. Connect the mainboard via a USB Type-C cable with the computer and enable the 12  V power supply for the mainboard. If the FT2232H-56Q chip is recognized correctly by the computer, two COM ports are opened. Open Xilinx Vivado 2023.2 and execute the command “program_ftdi −write −ftdi FT2232H −serial 0ABC01 −vendor “my vendor” −board “my board” −desc “my product desc”” via the tcl-console. If this has worked correctly, after a power cycle of the motherboard only one COM port remains open. Then, in Xilinx Vivado 2023.2 select “Flow → Open Hardware Manager”. Click on “Open target” and select “Auto Connect”. If the Zynq is recognized, an instance with the name “xc7z030_1” is displayed. Right click on “xc7z030_1” and select “s25fl256s-3.3v-qspi-x4-single” in the “Add Configuration Memory Device” section and press “OK”. Select the provided “BOOT.BIN” as the configuration file and the first stage bootloader file “fsbl.elf” for the Zynq FSBL. Set the “Bin Offset” to “0”, enable “Erase”, “Program” and “Verify” and press “OK”. As the firmware image is now stored in the QSPI flash, the dip switch SW1 must be set to QSPI mode according to the marking on the silkscreen. When the SoC module is restarted via the reset button S1, the Zynq now boots via the non-volatile QSPI flash.

### 6.4 Data acquisition system assembly

Once the printed circuit board assembly has been successfully completed, the data acquisition system must be assembled. Connect the mainboard with the ADC board via SFP + modules and the optical transmitter/receiver including all optical fibers. For network communication, connect the RJ-45 connector via a RJ-45 cable with either a network switch or direct with a computer. Furthermore, if the debug interface is used, connect the USB Type-C connector to a computer. Lastly, connect the mainboard with a 12  V power supply and the ADC board with an isolated power supply between 5  V and 42  V.

### 6.5 Debugging

The data acquisition system has several status LEDs and a USB interface to simplify debugging. Both the mainboard and the ADC board have status LEDs (D2, D3 and D7 on the mainboard and LED D4 on the ADC board) that light up when the supply voltage is present. In addition, there are several LEDs that indicate the status of the optically isolated data transmission between the mainboard and the ADC board. If the SFP + module is correctly plugged into the SFP + cage, LED D4 on the mainboard and LED D1 on the ADC board light up green, indicating a valid contact. After resetting the mainboard, the LED D6 on the mainboard and D3 on the ADC board start flashing, indicating the synchronization process of the JESD204 interface. When the LEDs stop flashing and stop lighting up, the JESD204 interface has been successfully synchronized. To debug the firmware, the serial interface from USB to UART can be used, as already described by Kobelt et al. [18]. The serial interface must be configured with a baud rate of 115200, no parity, 1 stop bit and no flow control.

## 7 Operation instructions

This work is a significant enhancement of the open-source isolated data acquisition with trigger pulse generation for ion mobility spectrometry published by Kobelt et al. [18] regarding the sampling rate and the SNR of the data acquisition, which includes an innovative, low-cost optical isolation for data transmission. To enable the user to use the same software for both variants of isolated data acquisition, the newly developed high-speed data acquisition system presented here is fully compatible with the open source software “IMS Control” [19]. The only difference in the application is that the user can now choose between the four predefined sampling rates 12.5 MS/s, 10 MS/s, 5 MS/s and 4 MS/s as required by the application. To improve the SNR and avoid aliasing, the cut-off frequency of the low-pass FIR filter in the FPGA is adjusted to match the selected sampling rate. To provide the user all the information about the low-pass FIR filter, the sets in the “IMS Control” have now been extended to include the cut–off frequency and the pass frequency of the low-pass FIR-filter, as well as the resulting SNR of the data acquisition depending on the selected sampling rate. An additional “Pipeline Delay” set has also been added to “IMS Control”. This pipeline delay is the delay of the entire data pipeline, summing up the ADC delay, the delay of the optical data transmission and the delay of the low-pass FIR filter. The pipeline delay is compensated internally in the FPGA. Furthermore, it should also be emphasized that the new high-speed data acquisition system has only one channel. Since the operation does not differ from Kobelt et al. in terms of acquisition parameters, ADC calibration, pulse generation and trigger modes, reference is made here to the publication by Kobelt et al. [18].

## 8 Validation and characterization

• Pipeline latency of 2.22 µs is compensated in FPGA
• Four different low-pass FIR filter for improving SNR and antialiasing
• Low-pass filtering and downsampling enables SNR of up to 87.5 dB and sampling rate of up to 12.5 MS/s
• Isolation tested of up to 13  kV, more possible due to the use of optical fibers for data transmission

### 8.1 Latency compensation

The latency of this data acquisition is the time span by which the measured voltage is delayed by the data acquisition pipeline. The delay of the data acquisition pipeline consists of the ADC delay, the delay of the optical data transmission and the delay of the low-pass FIR filter. The data acquisition for an IMS spectrum is started in the FPGA by an internal trigger signal and the data point that is in the downsampling unit at this time is saved inside the RAM. Due to the latency of the data acquisition pipeline, the data points arrive at the downsampling unit with a delay, causing old data points to be incorrectly assigned to the current IMS spectrum. However, if this latency is known and constant, it can be compensated for by delaying the internal trigger that starts the data acquisition by exactly the time span of the latency. Consequently, the latency must be determined by measurements.

Therefore, two synchronous pulses (with logic levels adapted to the circuit) were generated using an arbitrary waveform generator (Agilent, 81150A), whose output is connected to the ADC input and the trigger input (direct trigger mode) of the data acquisition system.

**Fig. 6:**
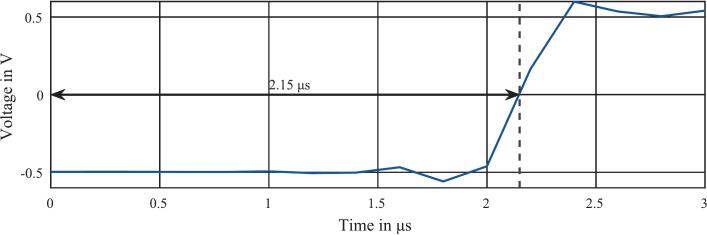
Spectrum of the two synchronous pulses connected to the ADC input and the trigger input.

**Fig. 7:**
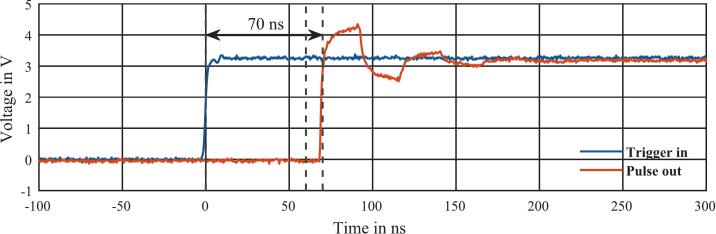
Measurement of the trigger input pulse and the corresponding pulse channel output.

As shown in Fig. 6, the delay of the pulse caused by the latency is 2.15 µs. It should be noted that the measured latency of the data acquisition is additionally delayed by the latency of the trigger in this measurement, so the latency of the trigger still has to be added. In order to determine the latency of the trigger, a pulse is generated with an arbitrary waveform generator (Agilent, 81150A) and connected to the trigger input (direct trigger mode) and connected to an oscilloscope (Keysight, InfiniiVision MX0X6004A). An output pulse is then generated instantly from the received trigger pulse in the FPGA via a pulse channel, which is also connected to the oscilloscope. Since the pulse generation in the FPGA runs immediately for the synchronization of the trigger, the time difference between the pulses in the oscilloscope corresponds exactly to the trigger latency. As shown in Fig. 7, the latency of the trigger (direct trigger mode) is between 60  ns – 70  ns. The interval of 10  ns results from the synchronization of the trigger pulse to the internal FPGA clock with a clock frequency of 100  MHz. However, this synchronization-related variation in delay can be interpreted as constant regarding arrival times of the ions of several 100 µ s in HiKE-IMS. Adding constant trigger delay to the previously measure delay, an overall latency of 2.22  µs for the complete data acquisition pipeline was determined and compensated by a constant delay of the internal trigger signal of 2.22  µs in the FPGA. This latency is displayed in the “IMS Control” as the “Pipeline Delay” set.

### 8.2 Increasing the SNR with low-pass filtering and downsampling

**Fig. 8:**
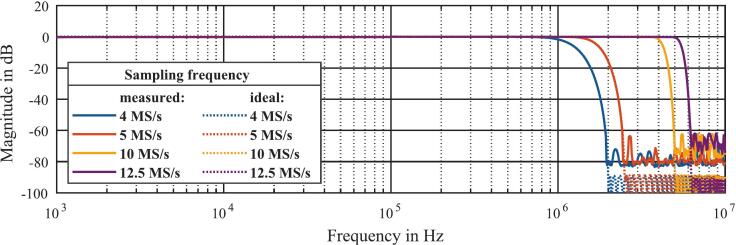
Comparison of the measured and ideal magnitude response of the four predefined low-pass FIR filters.

Since the application does not require the full 100 MS/s, the ADC data in the FPGA can be downsampled to the four adjustable sampling rates of 4 MS/s, 5 MS/s, 10 MS/s and 12.5 MS/s. In order to increase the SNR and avoid aliasing effects, four low-pass FIR filters adapted to the sampling rates were implemented in the FPGA. Symmetrical filter coefficients ensure that the low-pass FIR filters have a constant group delay for all frequencies, so the input signal is not deformed. The four predefined filters are designed with regard to their transfer function to have an attenuation of at least –88  dB at the cut-off frequency, which is, according to the Nyquist frequency, half of the corresponding sampling rate. These conditions result in a specific cut-off frequency for each low-pass FIR filter. In order to validate the function of the implemented low-pass FIR filters, the magnitude response was measured. Therefore, a frequency sweep was performed with an arbitrary waveform generator (Rigol, DG4062) and the peak-to-peak voltage within one spectrum was determined and normalized to the stimulus voltage. A comparison between the measured and ideal magnitude response of the four low-pass FIR filters is shown in Fig. 8. As can be seen, the measured and ideal magnitude response curves match very well over the complete frequency range. Due to the noise of the experimental setup within the passband of the low-pass FIR filter, the measured magnitude response is significantly higher than the ideal magnitude response. Since the noise is calculated from the integration of the noise density from 0  Hz to the stop frequency, the noise increases with increasing cut-off frequency, as shown an Fig. 8.

In order to validate the improvement of the SNR by the different low-pass FIR filters, the SNR was measured. To determine the noise of the data acquisition and to minimize the noise from the measurement setup, the two differential inputs were shorted against each other. The SNR depends on the Root-Mean-Square (RMS) value of the noise voltage $V_{noise,rms}$ and the RMS value of a harmonic signal that extends peak-to-peak over the full-scale voltage range of the ADC. The RMS value of the noise voltage $V_{noise,rms}$ corresponds to the standard deviation of the input voltage $\sigma_{rms}$, according to equation (3).

(3) $$V_{noise,\;rms}=\sigma_{rms}$$

The RMS value of a harmonic signal $V_{\mathrm{sig},rms}$, which extends peak-to-peak over the full-scale voltage range of the ADC with the voltage $V_{ADC,FS}$ is calculated using equation (4).

(4) $$V_{sig,rms}=\frac{V_{ADC,FS}}{2\sqrt{2}}$$

Thus, the measured SNR is calculated according to equation (5) for each of the four predefined sampling rates and the corresponding low-pass FIR filter.

(5) $${\mathrm{SNR}}_{meas}=20∙\log_{10}\frac{V_{sig,rms}}{V_{noise,\;rms}}=20∙\log_{10}\frac{V_{ADC,FS}}{2\sqrt{2}\sigma_{rms}}$$

**Fig. 9:**
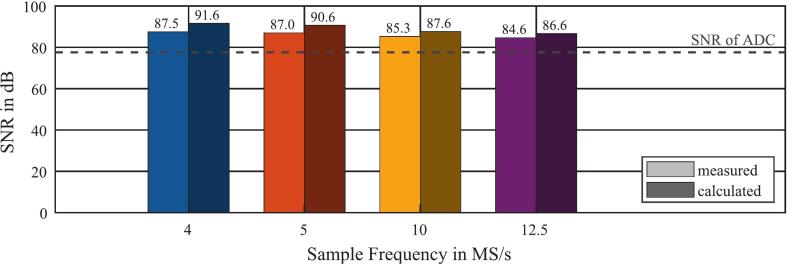
Comparison of the measured SNR and the calculated SNR for the four predefined sampling rates.

According to equation (5), $\sigma_{rms}$ was measured for each of the four sampling rates and corresponding ${\mathrm{SNR}}_{meas}$ was calculated with a full-scale voltage of the ADC with $V_{ADC,FS}=2.25V$. The comparison between the measured ${\mathrm{SNR}}_{meas}$ (displayed bright) and the calculated ${\mathrm{SNR}}_{calc}$ (displayed dark), increased by oversampling and low-pass filtering and calculated for each of the four predefined sampling rates according to equation (2), is shown in Fig. 9. The SNR of the ADC of 77.6 dB (12,6 bit ENOB), is also plotted as a reference.

For example, the SNR of the ADC of 77.6  dB (12.6 bit ENOB) is increased by up to 9.9  dB to a ${\mathrm{SNR}}_{\mathrm{meas}}$ of 87.5 dB (14.2 bit ENOB) at a sampling rate of 4 MS/s by downsampling and low-pass filtering. For the other sampling rates, the SNR is also significantly improved by low-pass filtering in combination with downsampling. Despite a basically good agreement between ${\mathrm{SNR}}_{\mathrm{meas}}$ and ${\mathrm{SNR}}_{\mathrm{calc}}$, it is noticeable that the ${\mathrm{SNR}}_{\mathrm{meas}}$ is always slightly lower than the ${\mathrm{SNR}}_{\mathrm{calc}}$. To calculate the improvement of the SNR by low-pass filtering in combination with downsampling, the simplified assumption was made that the entire noise consists only of quantization noise, which is evenly distributed over the frequency range. All other possible sources of noise, such as thermal noise or power supply noise, are not considered in this calculation. Since these additional noise sources increase the overall noise power, the ${\mathrm{SNR}}_{\mathrm{meas}}$ must be slightly lower than the ${\mathrm{SNR}}_{\mathrm{calc}}$. This limits the maximum ${\mathrm{SNR}}_{\mathrm{meas}}$ that can be achieved. In summary, low-pass filtering in combination with downsampling can be used to trade decreasing sampling rate for increasing SNR. This technique was successfully implemented and validated here.

### 8.3 HiKE-IMS spectrum

**Fig. 10:**
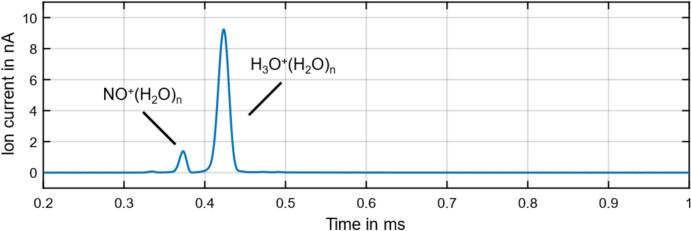
HiKE-IMS spectrum at 20.4 mbar with a corona ionization source operating at a reduced electrical field strength of 40 Td in the reaction region and a reduced electrical field strength of 100 Td in the drift region (1024 averages of spectra sampled with 10 MS/s).

Finally, to demonstrate the functionality of the new isolated high-speed data acquisition system presented here, an IMS spectrum was recorded using a HiKE-IMS, as shown in Fig. 10.

The HiKE-IMS has a corona ionization source and is operated at a pressure of 20.4 mbar. With a reduced field strength in the reaction region of 40 Td, the water-clustered reactant ions NO^+^(H_2_O)_n_ and H_3_O^+^(H_2_O)_n_ are formed [28]. To demonstrate the capabilities of the high-speed data acquisition system, the HiKE-IMS was operated with a reduced field strength of 100 Td in the drift region, requiring high drift voltage of 13  kV and resulting in very sharp peaks. Thus, a high isolation strength of 13  kV is required.

For this parameter setting, the NO^+^(H_2_O)_n_ and H_3_O^+^(H_2_O)_n_ have a full-width-at-half-maximum (FWHM) of around 10  µs. This means, that the previous data acquisition system of Kobelt et al., which provides a sampling rate of 250 kS/s [18] respectively a sampling interval of 4  µs is not capable to sufficiently fast sample such IMS spectra. The enhanced high–speed data acquisition system presented here, however, provides a sampling rate of up to 12.5 MS/s respectively a sampling interval of 80  ns, which enables to sample even peaks with very low FWHM as present in HiKE-IMS. The spectrum shown in Fig. 10 was recorded at a sampling rate of 10 MS/s and results from the averaging of 1024 IMS spectra.

## CRediT authorship contribution statement

**Jannik Wuttke:** Writing – original draft, Visualization, Validation, Software, Methodology, Conceptualization. **Tim Kobelt:** Validation, Software, Conceptualization. **Jonas Winkelholz:** Writing – review & editing, Methodology, Conceptualization. **Martin Lippmann:** Writing – review & editing. **David Bailey:** Validation. **Stefan Zimmermann:** Writing – review & editing, Supervision, Funding acquisition, Conceptualization.

## Declaration of competing interest

The authors declare that they have no known competing financial interests or personal relationships that could have appeared to influence the work reported in this paper.
